# Supplementary figures and images for: SMN-deficient cells exhibit increased ribosomal DNA damage
Source: Life Sci Alliance. 2022 Apr 19;5(8):e202101145. doi: 10.26508/lsa.202101145 (PMC9018017; doi:10.26508/lsa.202101145)

## Source Data for Fig S6B

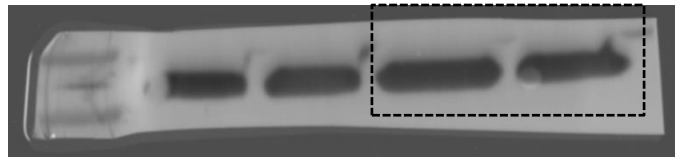

α-tubulin

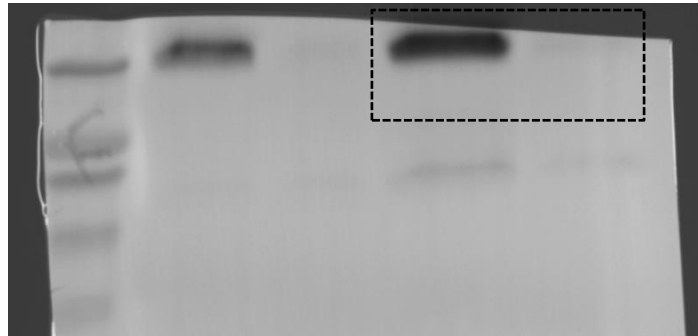

SMN

## Source Data for Fig S6D

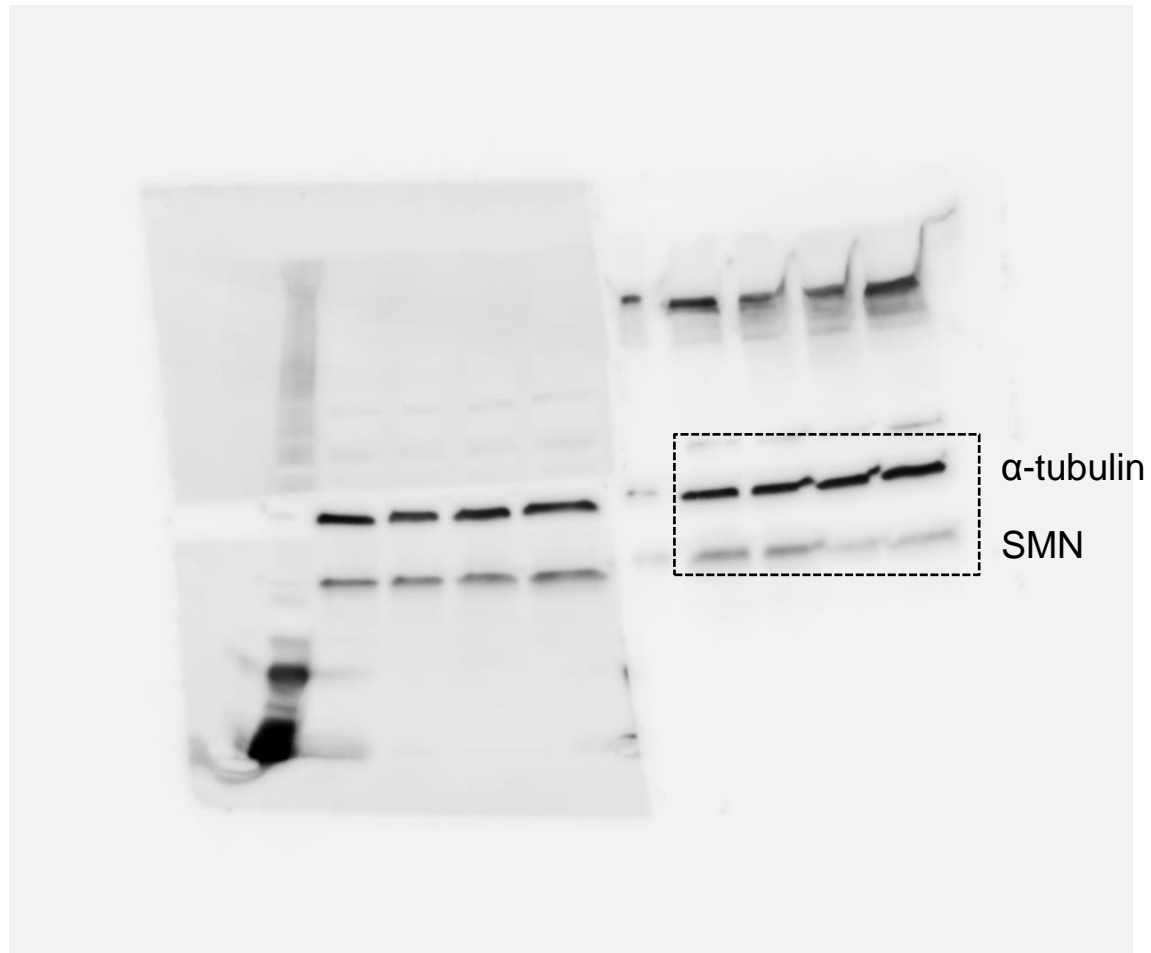

Supplement: Supplementary file 1 [file LSA-2021-01145_SdataFS6.pdf]

## Source Data for Fig S9

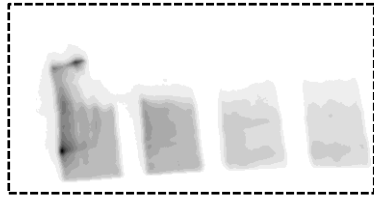

RNA pol II

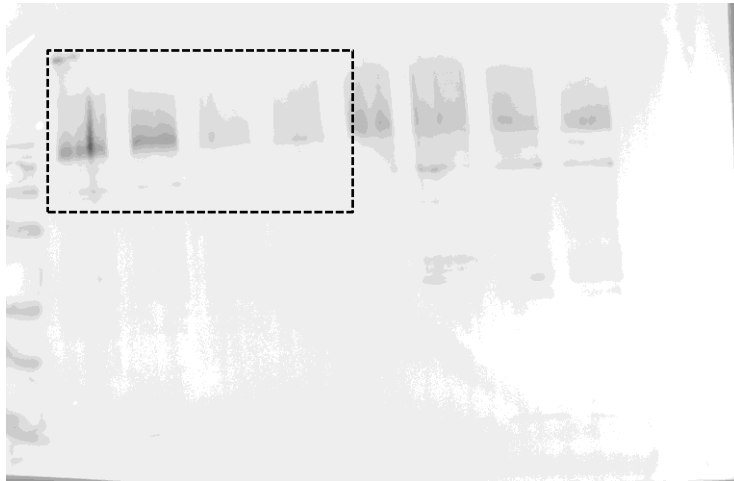

RNA pol I

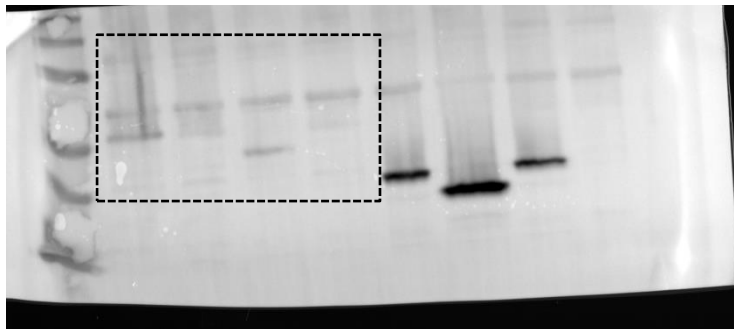

GFP

Supplement: Supplementary file 3 [file LSA-2021-01145_SdataFS9.pdf]
