# Supplementary material for: SMN-deficient cells exhibit increased ribosomal DNA damage
Source: Life Sci Alliance. 2022 Apr 19;5(8):e202101145. doi: 10.26508/lsa.202101145 (PMC9018017; doi:10.26508/lsa.202101145)

Source Data for Fig 4A

RNA pol II

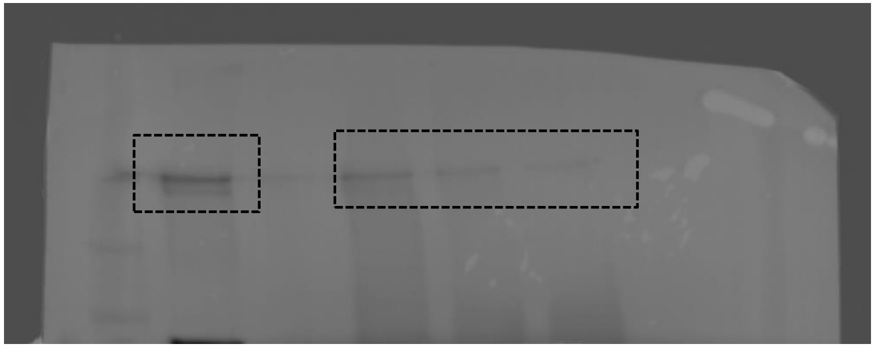

RNA pol I

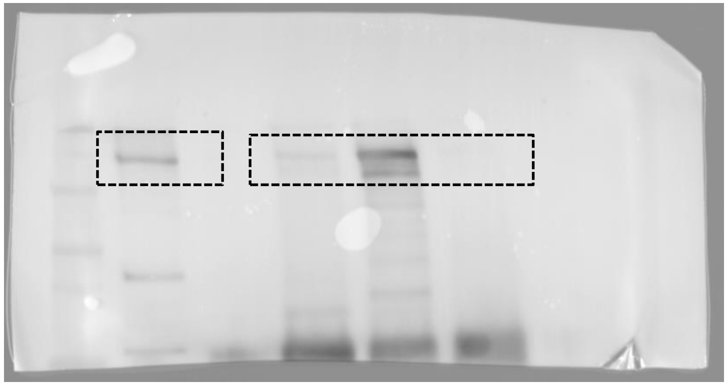

SETX

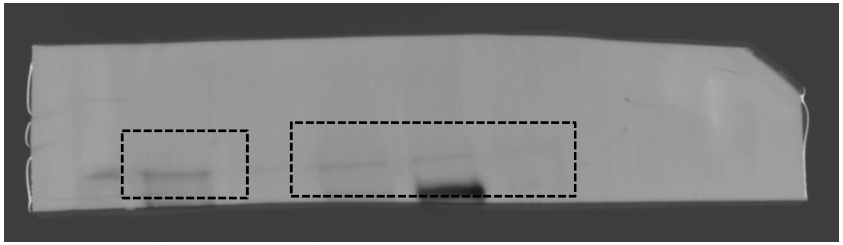

TOPO I

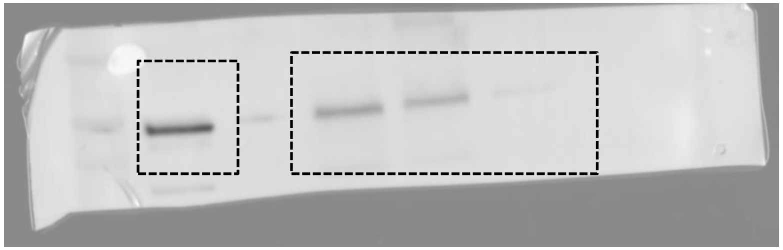

SMN

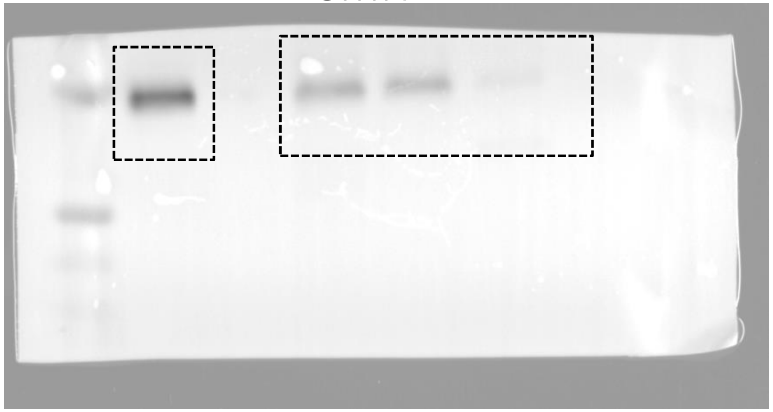

Source Data for Fig 4B

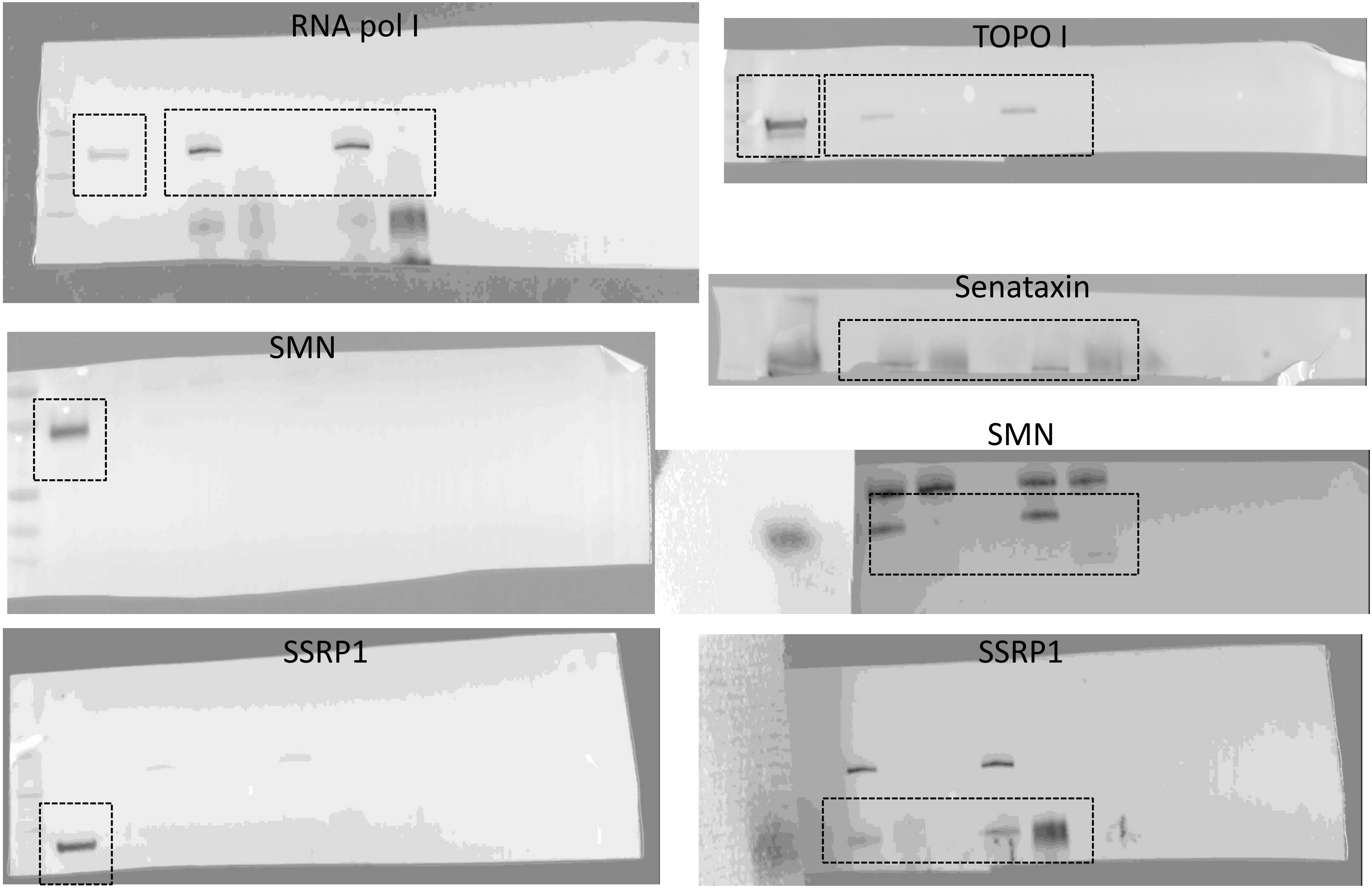

Source Data for Fig 4E

RNA pol II

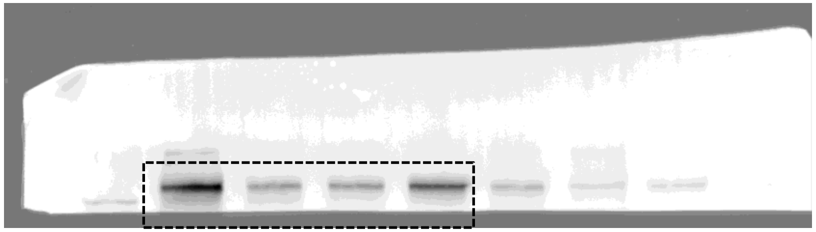

RNA pol I

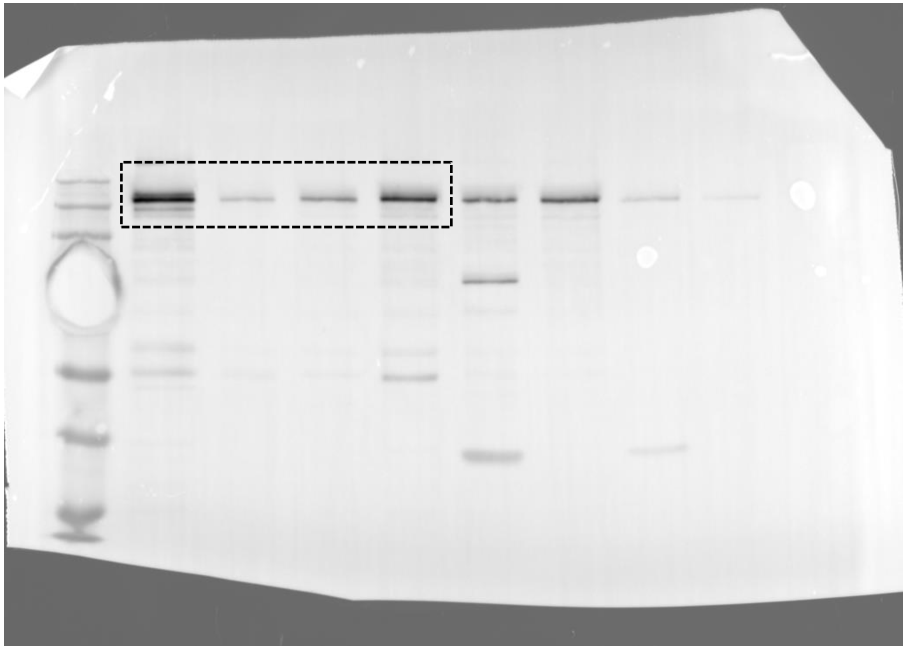

GFP

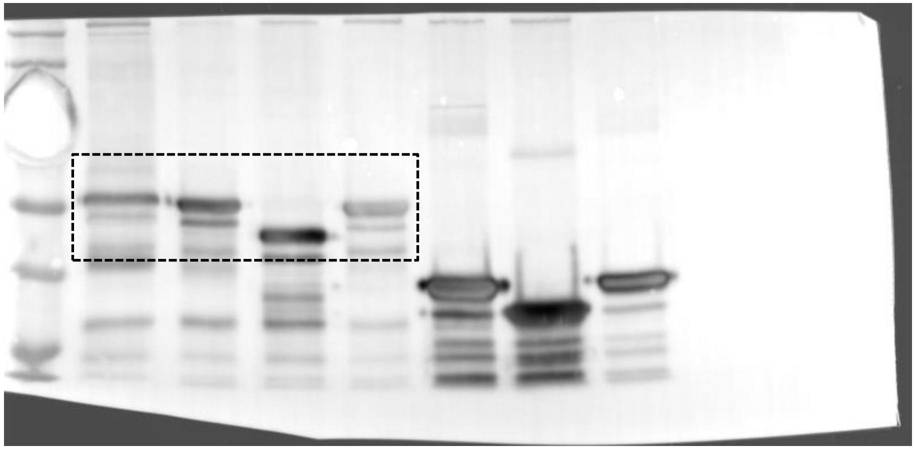

Supplement: Supplementary file 2 [file LSA-2021-01145_SdataF4.pdf]
